# Drugs and Constipation in Elderly in Nursing Homes: What Is the Relation?

**DOI:** 10.1155/2012/290231

**Published:** 2012-02-29

**Authors:** Gunvor S. Fosnes, Stian Lydersen, Per G. Farup

**Affiliations:** ^1^Department of Medicine, Innlandet Hospital Trust, 2819 Gjøvik, Norway; ^2^Unit for Applied Clinical Research, Department of Cancer Research and Molecular Medicine, Faculty of Medicine, Norwegian University of Science and Technology, 7491 Trondheim, Norway; ^3^Department of Research, Innlandet Hospital Trust, 2381 Brumunddal, Norway

## Abstract

*Introduction*. Constipation is a common adverse drug reaction. *Objective*. Study associations between drugs and constipation in nursing home residents. *Design*. Cross-sectional study. *Material and Methods*. Nursing home residents above 60 years of age were included. Demographics, diet, physical activity, activity of daily living, nutritional status, use of drugs, and diseases were recorded. Constipation was defined as functional constipation or constipation-predominant IBS according to the Rome III criteria and/or regular use of laxatives. Drugs were classified according to the Anatomical-Therapeutic-Chemical Classification System (ATC), and anticholinergic effect was noted. *Results*. In all, 79 men and 188 women with a mean age of 85.4 (SD 7.1) years were included. The prevalence of constipation was 71.5%. Use of drugs in general, including polypharmacy, was not associated with constipation. Reduced activity of daily living (OR = 0.71, 95% CI : 0.60–0.84, *P* < 0.001), *other antidepressants* (N06AX) (OR 3.08, 95% CI : 1.09–8.68, *P* = 0.03), and *benzodiazepine derivatives* (N05BA) (OR = 2.80, 95% CI : 1.12–7.04, *P* = 0.03) were significantly associated with constipation; drugs with markedly anticholinergic effect (OR = 3.7, 95% CI : 0.78–17.53, *P* = 0.10), *natural opium alkaloid* (N02AA) (OR = 5.01, 95% CI : 0.95–25.94, *P* = 0.06), and *propionic acid derivatives* (M01AE) (OR = 7.00, 95% CI : 0.75–65.08, *P* = 0.09) showed a trend. *Conclusion.* In elderly with constipation, focus should be on specific groups of drugs and nonpharmacological factors, not on drugs in general.

## 1. Introduction

Chronic functional constipation affects 17–40% of the elderly and reduces quality of life [1–4]. In nursing homes, the prevalence of constipation is in the order of 44–74% [4–7].

Gastrointestinal symptoms including constipation are common adverse drug reactions [8]. Drugs have been reported as one of the most important causes of constipation, in addition to insufficient intake of fluid and dietary fibre, reduced activity of daily living, lack of exercise, and diseases such as neurological and metabolic disorders [5–7, 9]. 

In general, the use of drugs is high in elderly in nursing homes [10–13]. Frail elderly with chronic diseases, altered pharmacokinetics and pharmacodynamics, and use of several drugs with anticholinergic effects and in part unknown interactions appear to be at high risk of side effects of drugs [8, 10–12, 14–16].

This cross-sectional study aimed at finding the impact of drugs on constipation in elderly in nursing homes.

## 2. Material and Methods

### 2.1. Study Design and Methods

In 2008-2009, this cross-sectional study was performed in nursing homes in the counties of Oppland and Hedmark, Norway. Registered and auxiliary nurses with good knowledge of the residents collected data from the medical records and got information from the residents and their next of kin. A blood sample was collected.

### 2.2. Study Population

Residents above 60 years of age living in nursing homes for more than 8 weeks and without organic gastrointestinal diseases that could cause constipation were included in the study.

### 2.3. Variables

The following variables were recorded: age, gender, weight, height, smoking habits, use of alcohol, somatic and psychiatric diseases, physical activity (walking steps/day, mobility), activity of daily living (Katz' Activity of Daily Living (ADL) index (score 0 (very dependent)–6 (independent)), nutritional status (Mini Nutritional Assessment (MNA score 0–30: <17 malnourished; 17–23.5 at risk of malnutrition; 24–30 normal nutritional status) [17], diet (fibre and amount and type of fluid), type of food (mashed food/soups, bread without crust, ordinary food), all use of drugs, a detailed description of the bowel function, continence for urine and faeces, dental status, signs of dehydration, and bedsore. A laboratory screen was performed.

Constipation was defined according to the Rome III criteria for functional constipation [18] except for insufficient criteria for irritable bowel syndrome because cognitive impairment made this information unreliable, and/or regular use of a laxative. Drugs were measured as use of drugs (yes/no) and the number of drugs (laxatives, dermatologicals, and topical preparations for eyes and ears were excluded). The drugs were grouped according to the Anatomical-Therapeutic-Chemical Classification System (ATC) at level four since drugs at this level probably have common adverse drug reactions [19]. In addition, anticholinergic effect (yes/no) was recorded as use of one or more drugs with markedly anticholinergic effect defined as level 3 according to Carnahan et al. [20]. Drugs for constipation (ATC-classes A06) and groups of drugs used by less than 10 residents were excluded from all analyses.

### 2.4. Statistical Analysis

Associations between constipation and the recorded variables were analyzed with Student's *t*-test, Mann-Whitney *U* test, Fisher's exact test, and Chi-square for trend. Three multivariable analyses (stepwise backward logistics regression) were performed with constipation as dependent factor and either number of drugs, drugs with markedly anticholinergic effect, or groups of drugs at ATC-level 4 with *P* ≤ 0.2 in bivariate analyses as independent variables. In addition, variables presumed to be associated with constipation (physical activity, intake of fibre, and fluid), and other variables associated with constipation with *P* ≤ 0.05 in the bivariate analyses were included in the analyses. The cut-off point for groups of drugs was set to *P* < 0.2 to avoid loss of any group of drugs since this was the primary aim of the study. The cut-off point for other variables was *P* < 0.05 to reduce the number of covariates in the calculations. Since a drug is highly associated with the disease under treatment, both the group of drugs and the disease were included if one of them was associated with constipation with *P* ≤ 0.2 in the bivariate analyses. The one with the lowest impact on the outcome in the multivariable analyses was removed. Age and gender were maintained in all analyses, and number of drugs and the anticholinergic effect were maintained in the respective analyses, independent of degree of significance. Other variables were removed one by one in the backward regression analyses until only variables with *P* < 0.1 remained in the equation. Foreseeable interactions were controlled for.

Multiple imputation for missing data was performed with all variables included in the analyses [21]. Two-sided *P* values ≤ 0.05 were regarded as statistically significant and *P* ≤ 0.10 as a trend. PASW Statistics 18 was used for the analyses. The power to detect a statistically significant difference (*α* ≤ 0.05) of 1.4 in the mean number of drugs used by subjects with and without constipation was 80%.

### 2.5. Ethics

This study was approved by the Regional Committee for Medical and Health Research Ethics, Central Norway, and by Privacy Ombudsman for Research at Oslo University Hospital and performed in accordance with the Declaration of Helsinki. All residents or their next of kin gave written informed consent to participate. Residents giving consent themselves were informed by the nursing staff. Next of kin or legally acceptable representative to residents unable to provide informed consent were given oral and written information during a visit to the nursing home or by post and phone before giving informed consent.

## 3. Results

**Figure 1:**
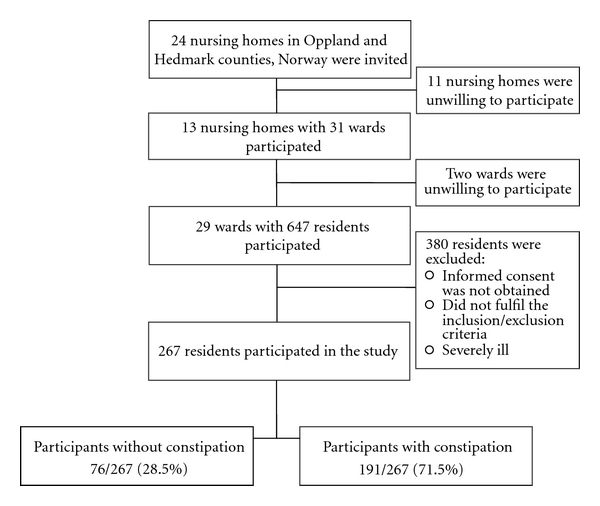
A flow chart of the participants in the study.

Out of 24 invited nursing homes, 13 participated. In the participating nursing homes, 267 out of 647 residents (41.3%) participated in the study.  Figure 1 shows the selection of the subjects, and Tables 1 and 2 show the characteristics of the residents and their use of drugs. The prevalence of constipation was 71.5%. The mean number of drugs used by the residents was 6.0 (SD 3.2, range 0–20); 17 (6.4%) used drugs with markedly anticholinergic effects.

Tables 1 and 2 also give comparisons between residents with and without constipation. Neither use of drugs, number of drugs, nor use of drugs with anticholinergic effects was statistically significantly associated with constipation. *Other antidepressants* (N06AX) was significantly associated with constipation, and *vitamin B12* (B03BA) and *thyroid hormones* (H03AA) with less constipation.

Table 3 gives the independent predictors for constipation (multivariable analyses). Neither number of drugs nor drugs with anticholinergic effect was significantly associated with constipation; drugs with anticholinergic effect showed a trend. *Other antidepressants* and *benzodiazepine derivatives* (anxiolytics) (N05BA) were significantly associated with constipation; *natural opium alkaloids *(N02AA) and *propionic acid derivatives *(M01AE) showed a trend. *Vitamin B12* and *thyroid hormones* were associated with less constipation. Analyses performed on original and imputed data did not differ in principal.

## 4. Discussion

Constipation was more prevalent in this study (71.5%) than in most corresponding studies [4–6]. Comparisons of prevalence rates are, however, difficult because the definitions of constipation vary. In this study, a combination of use of laxatives and the Rome III criteria for functional constipation and constipation-predominant IBS was used. Patients using laxatives regularly are supposed to have constipation although they do not fulfil the Rome criteria, and the two Rome III groups (functional constipation and constipation-predominant IBS) are difficult to distinguish in subjects with reduced cognitive functions and are probably not distinct groups and could be merged [22]. This definition is sensible in this study population. The validity of the Rome III criteria for constipation has, however, been questioned, but no other instrument is available [23].

### 4.1. Drugs and Constipation

The main finding was that use of drugs in general, including polypharmacy, was not significantly associated with constipation in this nursing home population with a high prevalence of constipation and polypharmacy. Most studies, reviews, and guidelines focus on drugs and polypharmacy as risk factors for constipation in elderly [4, 5, 7, 9, 14, 24].

The prevalence of use of drugs with markedly anticholinergic effect was three times higher in subjects with constipation than in those without. The constipating effect of drugs with anticholinergic effect has been reported in several studies and in reviews, and the nonsignificant finding in this study is probably due to low power since rather few subjects used this type of drugs [4–7, 9, 25].

A few groups of drugs were associated with constipation. *Other antidepressants,* which have also been associated with constipation in other studies, and *benzodiazepine derivatives* were significantly associated with constipation [5, 26]. *Propionic acid derivatives* (M01AE) and *natural opium alkaloids* (N02AA) showed a trend; the ORs were 7 and 5, respectively. The high ORs show that the prevalence of constipation was much higher in users than nonusers of these drugs and the lack of statistical significance was probably a type II error due to few users. The constipating effect of these drugs is known from several studies and reviews [4, 5, 7, 9, 26].

To our knowledge, the significant associations between *Thyroxin hormones *and* vitamin B12 *and less constipation have previously not been described. However, thyroxin has been associated with diarrhoea [27].

### 4.2. Other Factors and Constipation

Reduced activity of daily living was highly statistically significantly associated with constipation. Inactivity and dietary factors are explanations of this association, which is reported also in other studies and reviews [4, 5, 7, 9, 28]. The associations between both diabetes and low intake of fluids seen in this study have also been reported in other studies [4, 5, 26, 28]. Constipation was, as expected, somewhat higher in women than men [1, 2, 26].

### 4.3. Strengths and Weaknesses

The participating rate was lower than desired. Exclusion of 11 out of 24 nursing homes probably occurred by chance. The low participation rate in participating nursing homes (41.7%) might have influenced the external validity. Informed consent was easier to obtain from residents able to provide informed consent themselves than from the next of kin. This might explain why the prevalence of dementia was somewhat lower than in some other studies. The most frail and dement residents might therefore have been underrepresented [5, 6, 11, 12]. However, the prevalence of cardiac disease, cerebrovascular disease, and depression were somewhat higher than reported in other studies, indicating in all a representative sample [5, 6, 12]. The definition of constipation, which included regular use of laxatives, might contribute to the high prevalence of constipation [5, 6, 11, 12]. Subjects on regular use of laxatives probably have constipation when treatment is stopped, but it could not be ruled out that some patients continue treatment after ended indication. Despite these limitations, the external validity is judged as satisfactory since all other variables (age, sex, mobility, etc.) were within the range described in other studies [5, 6, 11, 12].

More information than normally in clinical studies was based on information from the nurses, next of kin and the medical journals, and less from the residents themselves, since the majority of the participants had cognitive impairment. This might have reduced the validity of the data, but use of deputies was inevitable.

Groups of drugs were analyzed at ATC-level 4, which are drugs with similar chemical structure and uniform side effects. Analyses on ATC-level 5 would have resulted in too small groups, and ATC-level 3, or higher combine drugs with different side effects.

The power of the study allows the exclusion of an effect of drugs in general, including polypharmacy, on constipation. The study also indicates an unfavourable effect of drugs with markedly anticholinergic effects (OR 3.7). Some associations between groups of drugs and constipation with high OR but not statistically significant associations (*P* = 0.05–0.10) have been reported because the associations might be of clinical significance, and the lack of statistically significance might be due to low power (few users of these drugs). The constipating effect of some groups of drugs used by few subjects might have been missed.

## 5. Conclusion

Use of drugs in general, including polypharmacy, was not associated with constipation in elderly in nursing homes, but some specific groups of drugs were. Therefore, in elderly with constipation, focus should be on specific groups of drugs and not drugs in general. This study indicated that focus should be on *benzodiazepine derivatives*, *other antidepressants, natural opium alkaloid, propionic acid derivatives,* and drugs with markedly anticholinergic effects, in addition to nonpharmacological interventions like activity of daily living.

## Figures and Tables

**Table 1:** Characteristics of the participants and comparisons between participants with and without constipation. The results are given as mean (standard deviation (SD)), median (range), and number (proportion (%)).

Characteristics^1^	All participants *n* = 267	Participants with constipation *n* = 191	Participants without constipation *n* = 76	Statistics *P* value
Age (years)	85.4 (SD 7.1)	85.3 (SD 6.9)	85.6(SD 7.5)	0.69
Gender (female)	188 (70.4%)	139 (72.8%)	49 (64.5%)	0.19
Ability to give informed consent	98 (36.7%)	66 (34.6%)	32 (42.1%)	0.26
Functional constipation (Rome III) (*n* = 266)	95 (35.7%)	95 (50.0%)^2^	0	n.a.
Body mass index (kg/m^2^) (*n* = 251)	25.5 (SD 4.9)	24.5 (SD 5.3)	24.7 (SD 4.9)	0.72
Smoking (*n* = 263)	19/49/195	13/30/144	6/19/51	
Current/before/never	(7.2/18.6/74.1%)	(7.0/16.0/77.0%)	(7.9/25.0/67.1%)	0.22
Use of alcohol > once a month (*n* = 263)	39 (14.6%)	28 (14.9%)	11 (14.7%)	1.00
Walking steps/day (*n* = 184)	200 (0–5000)	139 (0–5000)	400 (0–5000)	0.02
Mobility	120/80/67	100/48/43	20/32/24	
Bedridden/walk indoors/walk outdoors	(44.9/30.0/25.1%)	(52.4/25.1/22.5%)	(26.3/42.1/31.6%)	<0.01
Intake of fluids (Glass/day)	9.9 (SD 2.9)	9.0 (SD 2.8)	10.1 (SD 3.0)	<0.01
Dietary fibre (gram/day)	15.6 (SD 6.4)	15.3 (SD 6.3)	16.4 (SD 6.6)	0.18
Katz' Activity of Daily Living ^3^ (*n* = 254)	2 (0–6)	1 (0–6)	3 (0–6)	<0.001
Mini Nutritional Assessment score (*n* = 197)	21.4 (SD 3.5)	19.6 (SD 3.9)	21.5 (SD 3.6)	0.001
Type of food (*n* = 266)	41/44/181	33/36/121	8/8/60	
Mashed/without crust/ordinary food	(15.4/16.5/67.8%)	(17.4/18.9/63.7%)	(10.5/10.5/78.9%)	0.04
Incontinence urine only (*n* = 266)	93 (35.0%)	73 (38.2%)	20 (26.7%)	0.09
Incontinence urine and faeces (*n* = 265)	124 (46.8%)	90 (47.4%)	34 (45.3%)	0.79
Bedsore/wound (*n* = 265)	32 (12.1%)	28 (14.7%)	4 (5.3%)	0.04
Number of diseases (*n* = 266)	5 (1–15)	5 (0–15)	5 (0–10)	0.85
Heart diseases (*n* = 265)	118 (44.5%)	82 (43.2%)	36 (48.0%)	0.50
Venous thrombosis, pulmonary embolism (*n* = 264)	20 (7.6%)	17 (8.9%)	3 (4.1%)	0.21
Stroke (*n* = 264)	91 (34.5%)	72 (38.1%)	19 (25.3%)	0.06
Depression/anxiety (*n* = 266)	131 (49.2%)	100 (52.4%)	31 (41.3%)	0.13
Dementia (*n* = 265)	148 (55.8%)	105 (55.0%)	43 (58.1%)	0.68
Diabetes (both I and II) (*n* = 266)	32 (12.0%)	20 (10.5%)	12 (16.0%)	0.22
Parkinson's disease (*n* = 266)	15 (5.6%)	10 (5.2%)	5 (6.7%)	0.77
Hypothyroidism (*n* = 266)	20 (7.5%)	9 (4.7%)	11 (6.7%)	0.001
S-creatinine (above reference values) (*n* = 254)	62 (24.4%)	37 (20.4%)	25 (34.2%)	0.02
Albumin (g/L) (*n* = 167)	38.9 (SD 4.1)	38.5 (SD 4.2)	39.5 (SD 3.7)	0.16

^1^Number of residents is given in brackets when data are missing.

^2^Use of laxatives regularly is included in the definition of constipation.

^3^Katz Index of Independence in Activities of daily living. (Score 0–6: 0 = very dependent; 6 = independent).

**Table 2:** Use of drugs by the participants and comparisons between participants with and without constipation. The results are given as proportion (%) if not otherwise indicated.

Drugs	All participants *n* = 267	Participants with constipation *n* = 191	Participants without constipation *n* = 76	Statistics *P* value
Use of drugs	98.5	98.4	98.7	1.00
Number of drugs (median with range)	6 (0–20)	6 (0–20)	6 (0–19)	0.90
Drugs with markedly anticholinergic effect	6.4	7.9	2.6	0.17
Laxatives regularly only	61.0	85.3	0^1^	—
Laxatives on demand only	5.2	0	18.4	—
Laxatives regularly and on demand	68.9	96.3	0^1^	—
Contact laxatives (A06AB)	28.5	39.8	0^1^	—
Osmotically acting laxatives (A06AD)	52.1	72.8	0^1^	—
Enemas (A06AG)	4.9	6.8	0^1^	—
Proton pump inhibitors (A02BC)	14.2	15.7	10.5	0.15
Insulin and analogues (A10A)	3.7	2.6	6.6	0.15
Potassium (A12BA)	7.9	7.9	7.9	1.00
Platelet aggregation inhibitors (B01AC)	39.7	41.4	35.5	0.41
Bivalent oral iron (B03AA)	13.9	14.7	11.8	0.70
Vitamin B12 (B03BA)	12.4	9.4	19.7	0.04
Organic nitrates (C01DA)	8.2	6.8	11.8	0.22
Sulphonamides, plain (diuretics) (C03CA)	36.0	37.2	32.9	0.57
Beta blocking agents, selective (C07AB)	17.2	15.2	22.4	0.21
Dihydropyridine derivatives (C08CA)	6.7	7.9	3.9	0.29
Angiotensin II antagonists^ 2^ (C09CA_DA)	4.9	3.7	7.9	0.20
HMD-CoA reductase inhibitors (C10AA)	12.7	11.5	15.8	0.42
Thyroid hormones (H03AA)	9.7	6.8	17.1	0.02
Antibacterials—systemic use (J01)	15.0	14.1	17.1	0.57
Propionic acid derivatives (M01AE)	4.9	6.3	1.3	0.12
Bisphosphonates (M05BA)	6.4	6.8	5.3	0.79
Natural opium alkaloids (N02AA)	6.7	8.4	2.6	0.11
Phenylpiperidine derivatives (N02AB)	4.9	6.3	1.3	0.11
Other antiepileptics (N03AX)	4.1	4.7	2.6	0.73
Benzodiazepine derivatives (N05BA)	19.5	22.5	11.8	0.06
Benzodiazepine related drugs (N05CF)	29.2	30.9	25.0	0.37
SSRI^ 3^ (N06AB)	31.5	30.4	34.2	0.56
Other antidepressants (N06AX)	15.7	18.8	7.9	0.03
Anticholinesterases (N06DA)	6.7	4.7	11.8	0.06
Phenothiazine derivatives (R06AD)	5.2	5.2	5.3	1.00

^1^ Use of laxatives regularly is included in the definition of constipation.

^2^Both plain and in combination with diuretics.

^3^Selective serotonin reuptake inhibitors.

**Table 3:** Variables associated with constipation. The analyses were performed with either number of drugs (Analysis 1), anticholinergic effect (Analysis 2), or groups of drugs (Analysis 3) as independent variables in backward logistic regression analyses.

Independent variables	Constipation					
	Analysis 1		Analysis 2		Analysis 3	
	OR (95% CI)	*P* value	OR (95% CI)	*P* value	OR (95% CI)	*P* value
Number of drugs^1^	1.04 (0.94–1.15)	0.43				
Drugs with markedly anticholinergic effect^1^ (*n* = 17)			3.69 (0.78–17.53)	0.10		
Benzodiazepine derivatives (N05BA) (*n* = 53)					2.80 (1.12–7.04)	0.03
Other antidepressants (N06AX) (*n* = 42)					3.07 (1.09–8.68)	0.03
Vitamin B12 (B03BA) (*n* = 33)					0.39 (0.16–0.93)	0.03
Levothyroxine sodium (H03AA) (*n* = 26)					0.27 (0.10–0.72)	0.01
Propionic acid derivatives (M01AE) (*n* = 13)					7.00 (0.75–65.08)	0.09
Natural opium alkaloids (N02AA) (*n* = 17)					5.00 (0.95–25.94)	0.06
Age^1^	1.00 (0.95–1.05)	0.94	0.99 (0.95–1.04)	0.70	1.01 (0.96–1.06)	0.78
Gender (female)^1^	1.66 (0.86–3.21)	0.13	1.53 (0.81–2.91)	0.19	1.80 (0.89–3.61)	0.10
Activity of daily living	0.73 (0.63–0.85)	<0.001	0.72 (0.63–0.84)	<0.001	0.71 (0.60–0.84)	<0.001
Intake of fluids (glass/day)	—	—	—	—	0.90 (0.81–1.01)	0.07
Hypothyroidism (*n* = 20)	0.19 (0.07–0.56)	<0.01	0.26 (0.09–0.71)	<0.01	—	—
Diabetes (*n* = 266)	—	—	—	—	0.35 (0.14–0.88)	0.02
S-creatinine^2^(*n* = 254)	0.54 (0.27–1.10)	0.09	—	—	—	—

Age and gender have been included in all analyses regardless of significance. ^1^Number of drugs and anticholinergic effect have been included in the respective analyses regardless of significance. ^2^Serum Creatinine above reference values (90 and 105 *μ*mol/L for women and men, resp.). —Has not been included in the final analyses.
